# Periodontitis, Low-Grade Inflammation and Systemic Health: A Scoping Review

**DOI:** 10.3390/medicina56060272

**Published:** 2020-05-30

**Authors:** Gennaro Cecoro, Marco Annunziata, Morena Tina Iuorio, Livia Nastri, Luigi Guida

**Affiliations:** Department of Medical and Surgical Specialties and Dentistry, University of Campania “Luigi Vanvitelli”, 80138 Naples, Italy; marco.annunziata@unicampania.it (M.A.); morenaiuorio@gmail.com (M.T.I.); livia.nastri@unicampania.com (L.N.); luigi.guida@unicampania.it (L.G.)

**Keywords:** periodontitis, low-grade inflammation, systemic diseases, inflammatory status, inflammatory markers

## Abstract

*Background and objectives:* Periodontitis is a multifactorial chronic inflammatory infectious disease in which an infection is necessary, but not sufficient, for development of the condition. Individual susceptibility strictly linked to the immune and inflammatory response of the organism must also be present. Low-grade inflammation (LGI) is a systemic status of chronic sub-clinical production of inflammatory factors. This condition represents a risk factor for many chronic diseases including diabetes, cardiovascular disease, cerebrovascular disease, neurodegenerative disease and cancer. This scoping review aims to clarify, summarize and disseminate current knowledge on the possible link between periodontitis, LGI and systemic health. *Materials and Methods:* PRISMA Extension for Scoping Reviews guidelines were followed. An ad-hoc created keyword string was used to search the electronic databases of PubMed/Medline, Embase, The Cochrane Library and ClinicalTrials.gov. A hand search of specialized journals and their reference lists was also performed. *Results:* 14 studies that respected eligibility criteria were selected and analyzed. There is emerging evidence of strong links between periodontitis, LGI and systemic health. On the one hand, periodontitis influences the systemic status of LGI and on the other hand, the systemic production of inflammatory factors affects periodontitis with a bidirectional connection. *Conclusions:* LGI and the subsequent onset of a systemic inflammatory phenotype can be considered the common substrate of many chronic inflammatory diseases including periodontitis, with multiple mutual connections between them. Understanding of the biological principles and mechanisms underlying such a complex interrelationship could lead to significant improvements in the field of personalized diagnostics and therapeutic protocols.

## 1. Introduction

Periodontitis is an inflammatory-infectious disease involving tooth-supporting tissues and represents the most common cause of tooth loss in adults of industrialized countries [1]. The prevalence of periodontitis in the population increases with age. Recent World Health Organization estimates suggest that periodontitis is found in 5–20% of middle-aged (35–44 years) adults in Europe, and up to 40% of older people (65–74 years) [2,3,4,5,6]. Such a disease, which may result in tooth loss, was the eleventh most prevalent global disease in 2016 [4]. Periodontitis is a bacterially induced inflammatory disease linked to the individual’s oral microbiota and immune system [7]. Periodontopathogenic bacteria, such as *Aggregatibacter actinomycetemcomitans* (Aa), *Porphyromonas gingivalis* (Pg), *Treponema denticola* (Td), *Tannerella forsythia* (Tf), *Prevotella intermedia* (Pi) and *Fusobacterium nucleatum* (Fn) were divided into complexes [8] according to their virulence. The more the bacteria accumulate in dental plaque, the more they penetrate the gingival sulcus and, as a result, the more they induce an intense local inflammatory response [9,10]. However, the microbial insult is not enough per se` to induce the onset of the disease [11,12]. The development of periodontitis is also due to an individual susceptibility linked to a genetic basis [13] and to lifestyles (especially smoking and poor oral hygiene) which allow bacteria to express their pathogenic potential [14]. A struggle between periodontal microbes and immunity takes place, with the intervention of innate immunity (e.g., macrophages, dendritic cells, natural killer cells, neutrophils) and adaptive immunity (e.g., B and T lymphocytes) with the consequent release of pro-inflammatory molecules (e.g., interferon-gamma, interleukin-17, tumor necrosis factor, interleukin-1, interleukin-6) and enzymes (e.g., collagenases such as matrix metalloproteinases) [15]. Inflammation acts as both a friend and foe. On the one hand, the inflammatory response is a form of defense from our body protecting against the invasion of bacteria inside the deeper tissues (such as bone). On the other hand, if the inflammation persists and is poorly regulated, it causes an irreversible destruction of the periodontium with typical signs of periodontitis such as periodontal pockets, attachment loss, gingival recessions, tooth mobility, tooth migration and tooth loss [16,17]. The dentogingival epithelial surface area, including any pocket epithelium in direct contact with subgingival biofilm, represents the interface through which local inflammation can affect systemic health [18]. Such an area, previously estimated to be approximately the size of the palm of an adult hand (about 70 cm^2^) [19], has since been resized to mean values between 8 and 20 cm^2^ in mild to severe periodontitis, with a maximum value of about 40 cm^2^ [18]. During periodontitis, locally produced pro-inflammatory mediators such as interleukin-1 (IL-1), interleukin-6 (IL-6), tumor necrosis factor alpha (TNF-α) and prostaglandin E2 (PGE2), may move into the systemic circulation and subsequently exert effects on distant organs and increase and/or perpetuate an inflammatory state [20]. In fact, compared to subjects with a healthy periodontium, patients with periodontitis have higher values of circulating white blood cells (WBC) and/or of systemic inflammatory parameters such as C-reactive protein (CRP), a protein produced by the liver as a response to an external insult [21]. These observations have generated the hypothesis that the local inflammation caused by periodontitis may extend to a systemic level influencing the subject’s inflammatory load [22] and vice versa, that the systemic inflammation may affect periodontal health. Recently, certain authors studying the contribution of inflammation to the most common chronic diseases such as diabetes, obesity, cardiovascular and neurological diseases, have introduced the concept of “low-grade inflammation” (LGI) [23,24,25]. LGI is characterized by a low-grade chronic systemic production of inflammatory factors. To date, LGI is recognized as a risk factor for a number of chronic diseases including cardiovascular, cerebrovascular and neurodegenerative diseases and cancer [26,27,28,29,30]. It has also been suggested that LGI may be able to increase the risk of insulin resistance and type 2 diabetes [31]. The assessment of LGI takes place through the evaluation of circulating biomarkers (e.g., CRP, fibrinogen) or cellular biomarkers (e.g., WBC and platelet counts) [32,33,34,35,36], even though it is a condition which has not yet been consistently defined or measured. Hypotheses have been made regarding the relation between periodontitis and LGI, because it seems that periodontitis may contribute to induce and/or to maintain the systemic state of LGI and, furthermore, LGI may also be a risk factor for periodontitis [37,38,39]. Interestingly, the concept of LGI could explain the existing link between periodontitis and certain systemic comorbidities such as cardiovascular disease and Parkinson’s disease [40]. The aim of this study is to conduct a scoping review to clarify, summarize and disseminate current knowledge on the topics of periodontitis, low-grade inflammation and systemic health, in order to identify key concepts, theories, sources of evidence and gaps in the literature on this subject and to assist in the elaboration of proposals for future research.

## 2. Materials and Methods

The present review follows the PRISMA (Preferred Reporting Items for Systematic Reviews and Meta-Analyses) [41] guidelines for Scoping Reviews (http://www.prisma-statement.org/Extensions/ScopingReviews). A literature search was carried out in May 2020 by two independent and calibrated reviewers (G.C., M.T.I.) in the database of the National Library of Medicine MEDLINE/PubMed, Embase, The Cochrane Library and a public registry for clinical trials (www.clinicaltrials.gov). The authors created and adopted an ad-hoc search string (“periodontitis” or “periodontal disease”) and (“low grade inflammation” or “low grade systemic inflammation”). Only studies in the English language were considered. No other filters were applied. Reviews of the literature, interventional studies (either randomized or non-randomized controlled clinical trials), observational studies (either analytical or descriptive), case series or case reports and preclinical studies (either in vitro or animal research) explicitly exploring any link between LGI, periodontitis and systemic health were considered. Conference abstracts and editorials were not considered. No restriction on age or number of patients, as well as follow-up duration was considered. A hand search was also conducted in the major international journals of periodontics. The reference lists of all original research and review articles identified to be relevant to the subject were scanned for possible additional studies. After selection from title and abstract analysis, all full texts were carefully read and analyzed for the eligibility criteria (inclusion/exclusion). Results and findings from each included study were qualitatively analyzed. The search strategy is reported in Table 1.

## 3. Results

**Figure 1:**
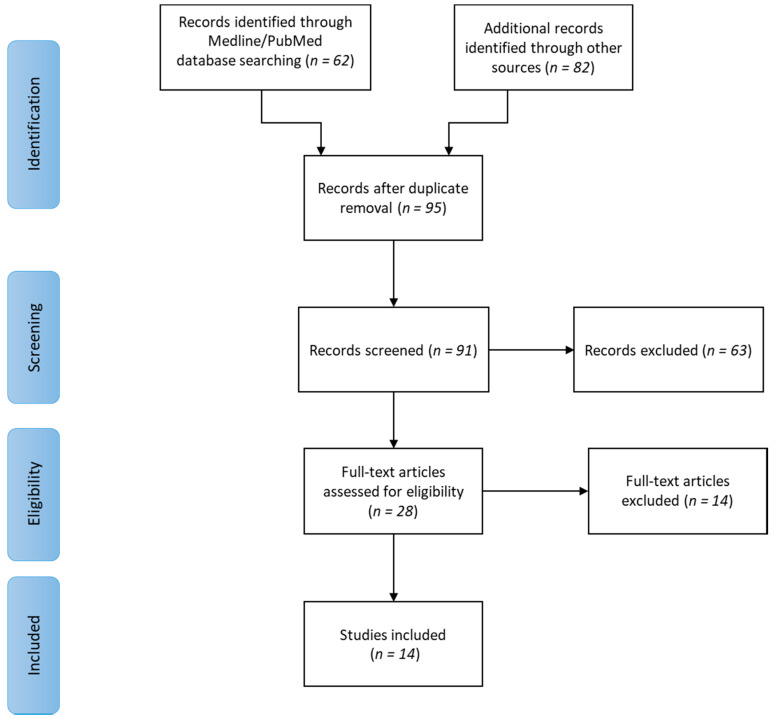
Flow diagram of the searching and selection process.

Initially, 144 scientific articles were found. After duplicate removal, 95 articles remained. Four of them were removed because they were not written in English (one in Danish, two in Chinese and one in German) and 63 articles were excluded after evaluation of title and abstract. The full texts of the remaining 28 articles were carefully read. As a result, 14 studies were excluded because they did not expressly identify a condition of LGI in correlation with periodontitis and systemic health. Finally, 14 studies were included in the present review. The flow diagram of the search and selection process is shown in Figure 1.

More specifically, three prospective cohort studies, one case-control study, one cross-sectional study, eight review studies and one animal study was selected. These studies concerned periodontitis, LGI and systemic conditions such as diabetes mellitus, atherosclerosis, cardiovascular diseases, obesity, cerebrovascular disease, coronary heart disease, endothelial dysfunction, chronic kidney disease, Alzheimer’s disease, Parkinson’s disease and systemic lupus erythematosus (SLE). In Table 2, the relevant findings from each study are summarized.

### 3.1. Periodontitis, LGI and Obesity

Obesity is correlated with periodontitis in four of the included studies [43,44,45,54]. Endo et al. [54] investigated the effects of periodontitis on the expression of pro-inflammatory cytokines in the liver and white adipose tissue (WAT) in Zucker rats. Twenty-four rats were divided into four groups of six rats each: lean rats without periodontitis (control group, CG), lean rats with periodontitis (periodontitis group, PG), obese rats without periodontitis (obesity group, OG), and obese rats with periodontitis (combination or mixed group, MG). At 4 weeks, the gene expression for CRP, IL-6 and TNF-α in the liver and CRP and IL-6 in the WAT of the MG was significantly higher than in each of the other three other groups. Serum TNF-α in the PG and OG was significantly higher than in the control group. Serum CRP and TNF-α in the MG was significantly higher than in each of the three other groups. The authors concluded that in the obese rat model, periodontitis increased the systemic LGI thereby upregulating the gene expression for hepatic levels of TNF-α and CRP and for IL-6 and CRP in the adipose tissue. In the lean rat model, periodontitis had little effect on the gene expression of pro-inflammatory cytokines in the liver and WAT. 

Meisel et al. [45] evaluated associations between adiposity and LGI with tooth loss in men and women. The authors postulate that sex-specific differences in the incidence of periodontitis and tooth loss may be related to different phenotypes of obesity and their associations with LGI. Follow-up data of 2.714 participants spanning five years from the cohort of Study of Health in Pomerania (SHIP), a population-based prospective cohort study in the northeast region of Germany [55,56,57], were analyzed for anthropometric measures, periodontitis, tooth loss, CRP and IL-6. Regression analyses were used to estimate the effect of obesity on tooth loss within sex strata. 

At the five year follow-up, with increasing waist circumference, the number of lost teeth increased both in women and men. Increased levels of CRP or IL-6 were significantly associated with the risk of losing teeth in men, whereas in women, this association was less distinctive. This study suggests that both adiposity and subclinical inflammation affect tooth loss, with distinct differences between men and women. Obesity as a risk factor for tooth loss may be modified by systemic markers of inflammation such as CRP and IL-6, especially in men. 

Gocke et al. [44] also investigated the relationship between periodontitis and LGI (which they link to atherosclerosis and cardiovascular diseases), and how such correlation might be modified by obesity status. In particular, they prospectively assessed the long-term impact of periodontitis, quantified by probing depth (PD) and clinical attachment level (CAL) measurements on systemic inflammation and quantified by fibrinogen serum levels and WBC counts on 2622 subjects from the SHIP with 5 and 11 year follow-ups. The authors used multilevel regression analyses adjusting data for common cardiovascular risk factors and stratifying analyses by abdominal obesity. They showed that positive associations between periodontal measures and inflammation markers were more pronounced in lean subjects compared to abdominally obese participants, in whom these associations were less pronounced or non-significant. The authors conclude that periodontitis promotes a chronic systemic LGI which may contribute, at least in part, to atherosclerosis, Such an effect, however, was masked in obese patients, probably because the adipose tissue produces inflammatory adipokines and cytokines, which may overwhelm the effects of periodontitis on systemic LGI. 

Pink et al. [43] aimed to demonstrate that LGI is a linking factor and a central hallmark of chronic diseases, such as obesity and diabetes mellitus. The study population comprised 1784 subjects from the SHIP with complete 11 year follow-up. Fibrinogen and WBC counts were measured as markers of inflammation. Periodontitis was assessed by PD, CAL and the Centers for Disease Control/American Academy of Periodontology (CDC/AAP) case definition. Multilevel regression analyses revealed significant coefficients for the impact of both inflammation markers on the percentage of sites with PD/CAL ≥3 mm. Increases in fibrinogen of about 1 g/L were associated with 3.0% and 2.7% more sites with PD/CAL ≥3 mm, respectively. Fibrinogen levels and WBC counts showed consistent long-term associations with PD and CAL. These results show that low-grade systemic inflammation is longitudinally associated with periodontitis in a consistent and dose-dependent manner and might represent one possible link for effects of obesity, diabetes or other chronic inflammatory conditions on the periodontium.

### 3.2. Periodontitis, LGI and Diabetes Mellitus

In addition to the study of Pink et al. [43], three review papers focused on periodontitis, diabetes and LGI [50,52,53].

Sima et al. [50] illustrate the bidirectional relationship that exists between diabetes mellitus and periodontitis. They report that poorly controlled diabetes correlates with higher prevalence, severity and progression rate of periodontitis compared with normoglycemic individuals, despite similar composition in subgingival biofilms. LGI is one of the possible mechanisms linking periodontitis and diabetes. Periodontitis raises the levels of pro-inflammatory mediators in serum, which may lead to insulin resistance and diabetes. In particular, a dose-response relationship between severity of periodontitis and plasma levels of TNFα, a cytokine known to promote insulin resistance, was found in adults with type 2 diabetes mellitus. Furthermore, the presence of severe periodontitis in diabetic patients gives them a higher risk of developing cardiovascular and renal diabetes-related complications compared to patients with no, mild, or moderate periodontitis. Periodontal therapy causes a significant reduction in hemoglobin A1c levels, but also a reduction in circulating inflammatory mediators (CRP, TNF, IL-6 and fibrinogen). Monocyte hyperactivity may be reversed in patients with diabetes mellitus by scaling and root planing resulting in reduced monocyte-derived TNFα, high sensitive CRP and sE-selectin.

The review of Santos Tunes et al. [52] focused on the immunobiological connection mechanisms between type 2 diabetes mellitus and periodontitis. Although there is extensive knowledge about the pathways through which diabetes affects periodontal status, less is known about the impact of periodontitis on the diabetes-related inflammatory state. This review attempts to explore the mechanisms by which periodontal infection can contribute to the LGI associated with diabetes and to discuss the impact of periodontal treatment on glycemic control in people living with both diabetes and periodontal disease. The low-grade chronic production of inflammatory factors and their direct flow from the periodontium to the systemic circulation in periodontal patients contributes to determinate a status of LGI, which may aggravate insulin resistance and adversely affect glycemic control. The authors affirm that, although conflicting, to some extent current evidence supports the hypothesis that periodontal treatment may improve insulin sensitivity and glycemic control by reducing periodontal inflammation and serum levels of cytokines and inflammatory markers. Since periodontal treatment seems to be equally able to lower hemoglobin A1c compared with other glucose lowering therapies, it may represent an alternative or adjunctive therapy to improve insulin sensitivity and glycemic control in diabetic patients with periodontitis.

Moutsopoulos et al. [53] highlight that several clinical studies demonstrate elevated systemic inflammation markers in periodontal patients. The leukocyte count has been shown to be slightly elevated in subjects with periodontitis compared to healthy subjects. This increase was proportional to the severity and extent of periodontitis, whereas the number of leukocytes decreased with periodontal therapy. Similarly, IL-6 levels have also been consistently shown to increase in relation to the extent of periodontitis. IL-6 is also a principal procoagulant cytokine and may also activate hepatocytes to produce acute phase reactants such as fibrinogen, plasminogen activator inhibitor 1 and CRP. In various studies included in the review of Moutsopoulos et al., periodontal patients had higher plasma levels of CRP compared to control groups, which ranged from 2 to 10 mg/L. These values are consistent with the presence of low-grade chronic inflammation. This condition may contribute to the pathogenesis of systemic inflammatory diseases such as cardiovascular diseases, atherosclerosis and diabetes. Periodontal therapy led to a decrease in serum CRP and cytokine levels and the most significant reduction was observed in patients with the highest baseline levels of CRP.

### 3.3. Periodontitis, LGI and Cardiovascular Disease 

Nibali et al. [46] investigated the association between severe periodontitis and an increase in inflammatory and metabolic risk factors for cardiovascular disease. They examined 302 patients with severe periodontitis and 183 healthy controls. Periodontitis patients were sub-classified according to the median number of pockets ≥5mm in higher extent (≥68 pockets) and lower extent (<68 pockets) clusters (median = 68). Periodontitis patients exhibited a higher number of WBC when compared with controls and, the higher the number of deep pockets, the higher the WBC count, in particular neutrophil and lymphocyte counts. The persistent periodontal inflammatory state might have a repercussion on the total number of circulating neutrophils because of increased bone marrow output or mobilization of the marginal granulocyte pool. 

Del Pinto et al. [47] recapitulate the determinants and consequences of the immune system dysfunction at an older age, with particular focus on the cardiovascular system. Periodontitis is mentioned by the authors as a main source of a chronic systemic LGI. In particular, the accumulation of advanced glycation end products (AGEs) during periodontitis is reported to trigger the cascade of pro-inflammatory signaling that, subsequently, activates redox-sensitive transcription factors responsible for endothelial cell hyper-permeability, vascular cell adhesion molecule-1 (VCAM-1) activation, chemotaxis and also cytokines/interleukins (TNF, IL-1, IL-6) release into the bloodstream. These circulating inflammatory mediators affect endothelial function, causing impaired vasodilation and alterations in vascular structure. The authors correlate such mechanisms with the systemic effects, as described in the literature, exerted by periodontal inflammation. In particular, periodontitis has been associated with a worse systolic blood pressure profile during antihypertensive therapy by about 2.3–3 mmHg and with higher odds of antihypertensive treatment failure. Furthermore, a history of periodontitis has been related to cerebrovascular disease, coronary heart disease, chronic kidney disease and eventually mortality. The authors speculate whether the control of potentially modifiable sources of LGI, such as periodontitis, may be a safe and effective preventive strategy against aging.

Similarly, Gurav [48] also confirms that periodontitis causes a systemic LGI status by production and spill over into the bloodstream of inflammatory markers which affect endothelial function. Specifically TNF-α and IL-6 are mentioned as being responsible for the reduction of NO production and, as a consequence, of endothelial dysfunction, which is considered a precursor to atherosclerosis and cardiovascular disease. In addition, this study affirms that periodontal therapy, reducing systemic inflammation, may improve endothelial function by the increase of NO bioavailability.

Shrihari [51] focuses his attention on the link between periodontitis and coronary heart disease. The author affirms that LGI caused by periodontitis is detectable through a very low increase of CRP levels which, if chronically maintained over time, can cause severe cumulative damage. The studies analyzed in Shrihari’s review show that periodontitis is associated with many cardiovascular risk conditions such as hypertension, acute myocardial infarction (AMI), stroke and increased carotid wall thickness. The more severe periodontitis is, the higher the cardiovascular risk. When considering the effects of periodontal treatment on inflammatory markers, all except one included studies which show a decrease in CRP levels after periodontal therapy. Patients showing a better response to periodontal therapy had a higher reduction in CRP levels together with a higher decrease of cardiovascular risk, confirming the mutual connection among all these aspects.

Holmstrup et al. [40] affirm that LGI is considered to be one of the most prominent mechanistic links between periodontitis and its comorbidities such as cardiovascular disease. The authors showed that periodontitis was more common in patients with previous AMI and among periodontal patients there was an increased risk of AMI. To explain the correlation between these two pathologies, the authors describe various proposed models. These include transfer of periodontal bacteria to atheromatous plaques, change of lipid metabolism, endothelial dysfunction, shared genetic risk factors and, focusing on LGI, the spillover in the bloodstream of proinflammatory cytokines from periodontal tissues, in particular IL-6 and TNF- α, which have been found augmented in periodontal patients. The authors also highlight how periodontal treatment may reduce some risk factors for atherosclerosis, including endothelial dysfunction, lipid parameters, glycated hemoglobin and biomarkers such as highly sensitive CRP and IL-6, especially in patients already suffering from coronary heart disease and diabetes mellitus. The authors indicate that severe periodontitis creates a higher risk for AMI and stroke and that periodontal treatment significantly reduces their incidence.

### 3.4. Periodontitis, LGI and Parkinson’s Disease

Holmstrup et al. [40] also focus their attention on Parkinson’s disease. Periodontitis is more common in patients with Parkinson’s disease [40,58] who also have a significantly higher rate of periodontal pockets ≥4 mm deep compared to patients without such a neurodegenerative disease (98.6% vs. 43.5%, respectively) [59]. This correlation might be attributable to the motor disability and cognitive changes that may impair oral hygiene maintenance. Interestingly, Parkinson’s disease seems to have an inflammatory component in its pathogenesis [60]. Although there is still no solid evidence, some studies indicate that systemic LGI induced by periodontitis may contribute to neural dysfunction at early stages of Parkinson’s disease.

### 3.5. Periodontitis, LGI and Alzheimer’s Disease

One included study [49] reports a possible link between periodontitis and Alzheimer’s disease. The author shows how pro-inflammatory cytokines released into the systemic bloodstream via the ulcerated periodontal pockets could compromise the blood brain barrier and gain access to the cerebral regions, thereby initiating the adverse repercussions that lead to neuronal damage consequent to the activation of microglial cells. Thus, systemic inflammation may serve as a connecting link between periodontitis and Alzheimer’s disease, although this causal relationship between the two pathologies still needs to be confirmed.

### 3.6. Periodontitis, LGI and Systemic Lupus Erythematosus 

Pessoa et al. [42] evaluated the reciprocal impact of periodontal subgingival microbiome on systemic lupus erythematosus and systemic inflammation. Circulating proinflammatory cytokines were upregulated in patients with SLE, inducing an LGI status. The presence of periodontitis, as well as of SLE, influenced oral microbiomes. The composition of the microbiome affects the levels of a wide range of cytokines. Thus, therapeutic control of dysbiosis changes of the oral microbiome due to periodontitis might reduce the systemic LGI burden, emphasizing the need to include periodontal diagnosis and treatment as part of the management of systemic diseases.

## 4. Discussion

Periodontitis and systemic health are mutually correlated, as periodontitis is influenced by systemic conditions and vice versa. It is significant that in the 2017 international classification of periodontal diseases, one of the most prevalent systemic diseases (uncontrolled diabetes mellitus [61]) has been considered a main modifying factor in the grading of periodontitis progression [62]. A growing body of literature also suggests an association with other systemic diseases such as cardiovascular disease, gastrointestinal and colorectal cancer, Alzheimer’s disease, as well as respiratory infection and adverse pregnancy outcomes. The biologic mechanisms underlying such correlations are still a matter for research [63]. In recent years however, the concept of a status of systemic LGI as a common background of several diseases including periodontitis, has increasingly attracted scientific attention. Following this hypothesis, periodontitis might contribute, at least in part, to the development and progression of chronic systemic diseases by consistently inducing a condition of LGI, a silent risk factor for many of them. Unfortunately, LGI is not still a well-defined conditio, and this scoping review was not able to find defined cut-off values of inflammatory markers sufficient for an LGI status. For instance, CRP values above 3 mg/L, but below 10 mg/L, have been reported as indicative of LGI [64,65,66]. However, neither of these values for this marker are univocally accepted, since CRP values lower than 3 mg/L have been reported as consistent with LGI [53]. In 2017, a composite score for LGI evaluation based on the assessment of plasmatic CRP levels and cellular biomarkers (leukocyte and platelet count and granulocyte/lymphocyte ratio) has also been proposed [67,68]. The adoption of such methods in future clinical studies could be a starting point to better grade this condition and to better evaluate its correlations with systemic disorders. 

To the best of our knowledge, this is the first PRISMA-driven scoping review specifically focused on the link between periodontitis, LGI and systemic health. A synthesis of the evidence in the literature following well defined criteria is still missing. It was decided to exclusively analyze studies that explicitly evaluated the correlation between periodontitis, LGI and systemic health in order to focus attention and collect information only about this emerging concept, thus excluding those studies where the authors analyzed systemic inflammatory conditions not expressly identified with an LGI status.

Periodontitis, due to its nature of infective inflammatory disease, involves the activation of the broad axis of innate immunity caused by the pathogenic action of subgingival microbiota through upregulation of proinflammatory cytokines from monocytes and polymorphonuclear leucocytes, including IL-1β, IL-6, IL-8, TNF-α and PGE2. Inappropriate secretion of these cytokines, in terms of either type or quantity, characterizes a dysregulated immune response that leads to loss of periodontal tissues. These locally produced cytokines move into the systemic circulation where they remain over time and may perpetuate an altered inflammatory status (e.g., increase insulin resistance and glucose levels). In this way, periodontitis may worsen already existing systemic diseases such as diabetes [50,52], and may even represent an important risk factor for the development of other non-communicable diseases (NCDs) such as osteoporosis, hypertension and angina pectoris [69]. 

**Figure 2:**
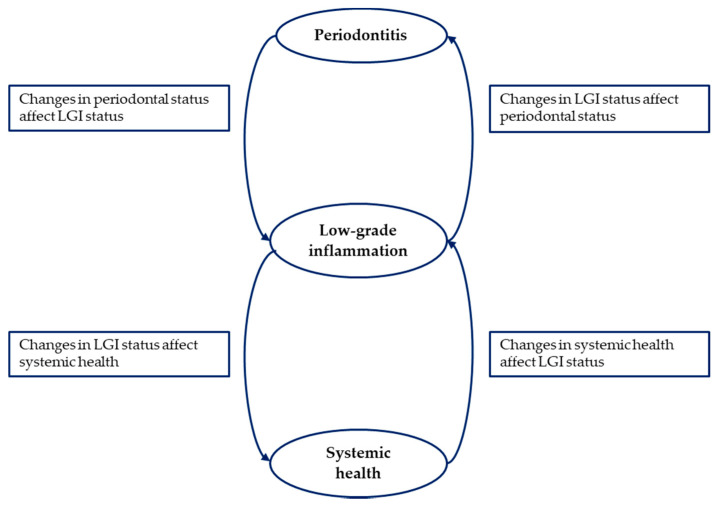
The central role of low-grade inflammation in the correlation between periodontitis and systemic health.

The synthesis of available data emerging from this scoping review confirms the strict connection existing between periodontitis, LGI and systemic health (Figure 2).

The systemic LGI status seems to be influenced by the composition of the oral microbiome [42], which changes as a consequence of periodontitis so that higher levels of circulating inflammatory markers have been described in periodontal patients compared to healthy subjects [46,50,51,52,53].

More specifically, as shown by Moutsopoulos et al. [53], CRP plasma levels are higher in periodontal patients compared to periodontally healthy subjects, with values, from 2 to 10mg/L, consistent with a status of systemic low-grade chronic inflammation. In addition, leukocyte counts and IL-6 levels are slightly higher in periodontal patients compared to healthy subjects. Similarly, the review by Santos Tunes et al. [52] shows increased levels of CRP, as well as IL-1, IL-6, TNF-α, PGE2 and fibrinogen in periodontal patients. Shrihari [51] affirms that periodontitis determines a status of LGI with a slight increase in CRP levels and, consequently, it may be an indirect risk factor for cardiovascular diseases. In line with these studies, Sima et al. [50] also report that periodontitis raises the levels of circulating pro-inflammatory mediators.

Endo et al. [54] show how periodontitis can cause an elevation in the number of polymorphonuclear leukocytes and, when this is combined with obesity, it may lead to higher gene expression of IL-6, TNF-α and CRP in the liver and in the adipose tissue, as well as to higher serum levels of TNF-α and CRP. 

Del Pinto et al. [47] and Gurav [48,49] highlight how periodontitis causes the release in the bloodstream of pro-inflammatory markers and, being a source of systemic LGI, it is able to affect systemic diseases such as CVD [47], endothelial dysfunction [48] and Alzheimer’s disease [49].

Systemic levels of inflammatory markers seem to be proportionally associated with the severity of periodontitis [43,44,45,46,50,51]. In particular, the study by Gocke et al. [44] highlights how increased PD and CAL values are directly correlated with LGI markers such as fibrinogen and WBC levels. Furthermore, Nibali et al. [46] show that the increasing number of periodontal pockets relates to higher WBC count. Conversely, the improvement of periodontal status and the healing of periodontal tissues after therapy have been shown to reduce the systemic levels of inflammatory markers [48,50,51,52,53] such as CRP, E-selectin and TNF- α, and also to help in the control of systemic conditions such as hyperglycemia [50,52], AMI and stroke [40] and endothelial dysfunction [48]. 

The analysis of the studies included in the present review also confirms the mutual and bidirectional link between periodontitis and systemic health by the involvement of LGI. 

In this sense, the prospective cohort studies of Pink et al. [43] and Meisel et al. [45] clearly show how periodontal health is influenced by the inflammatory state of the whole organism. In particular, higher systemic values of fibrinogen and WBC levels were associated with an increase of PD, CAL and the number of sites with PD and CAL ≥3 mm [43]. Furthermore, patients with increased levels of CRP, an inflammatory marker related to obesity as well as periodontal inflammation, were at higher risk of losing teeth compared to subjects with lower values of CRP [45]. 

It is noteworthy that CRP is a largely specific inflammatory marker and its values can suddenly vary based on multiple causes. Usually, repeated measurements of CRP are recommended to increase the reliability of the analysis [70,71]. None of the studies that focused on the relationship between this inflammatory marker and periodontitis included in the present scoping review refers to consecutive measurements of CRP. This aspect should be considered for future clinical studies with a similar aim in order to obtain more solid evidence.

## 5. Conclusions

The evidence summarized from the included studies supports the existence of a mutual correlation between periodontitis and systemic diseases mediated via LGI, although so far, there are no specifically designed clinical studies to confirm such a relationship. Furthermore, a specific case definition of LGI, with threshold values of defined inflammatory markers, is still lacking. Researchers are warmly encouraged to carry out well-designed observational clinical studies comparing systemic levels of LGI markers and periodontal clinical parameters in periodontal patients and periodontally healthy subjects with and without systemic co-morbidities. This will help to clarify the potential primary role of LGI in the mutual correlation between periodontitis and systemic diseases and will help obtain an univocal definition of the LGI condition. The understanding of the biological principles and mechanisms underlying such a complex interrelationship could lead to significant improvements in the field of personalized diagnostics and therapeutic protocols.

## Figures and Tables

**Table 1:** Search strategy.

Search Strategy
**Electronic databases:** MEDLINE/PubMed, Embase, The Cochrane Library, clinicaltrials.gov
**Search string:** “periodontitis” or “periodontal disease”; and “low grade inflammation” or “low grade systemic inflammation”
**Filter:** none
**Language:** English
**Inclusion criteria:** − Review articles, interventional and observational studies, pre-clinical studies; − No restriction on population characteristics, number of patients, age or systemic conditions; − Follow-up duration: any; − Explicit evaluation of the correlation between low-grade inflammation, periodontitis and systemic health
**Exclusion criteria:** conference abstracts and editorials
**Additional sources:** hand search of the major international journals of periodontics; reference lists of all screened studies

**Table 2:** Characteristics and relevant findings of the included studies.

Author, Year	Type of Study	Subjects, Groups	Co-Morbidity	Periodontal Outcomes Assessed	Inflammatory Markers Assessed	Relevant Findings
Pessoa et al., 2019 [42]	Cross-sectional study	91 subjects, 31 controls, 29 SLE-I, 31 SLE-A	SLE-I, SLE-A	PD, CAL, BOP	MIP-1-α, IL-1β, IP-10, IL-6, IL-8, IL-12p70, IL-17A, IFN-γ, GM-CSF, TNF-α, MIP-1β, IFN-α, MCP-1, P-Selectin, IL-1α, sICAM-1, E-selectin	Increased low-grade systemic inflammation was observed in SLE patients compared to controls. SLE and periodontitis influence microbiome composition. The levels of the various cytokines were associated with the presence and abundance of different microorganisms. By controlling oral microbiome-induced inflammation, the systemic LGI burden could be reduced.
Pink et al., 2015 [43]	Prospective cohort study	1784 subjects from SHIP	Obesity, diabetes mellitus	PD, CAL	Fibrinogen, WBC	At 11-year F-U, an increase in fibrinogen levels of 1 g/L was associated with an increase of 0.08 mm in mean PD, 0.10 mm in mean CAL, with 3% more sites with PD ≥3 mm, and 2.7% more sites with CAL ≥3 mm. For an increase in WBC levels of 10^9^ cells/L, the increase was of 0.03 mm for mean PD, of 0.05 mm for mean CAL, with 1.1% more sites with PD ≥3 mm, and 1.3% more sites with CAL ≥3 mm.
Gocke et al., 2014 [44]	Prospective cohort study	2622 subjects from SHIP	Obesity, atherosclerosis, CVD	PD, CAL	Fibrinogen, WBC	In lean subjects, the percentage of sites with PD/CAL ≥3 mm was associated with fibrinogen and WBC levels. At the 11-year F-U, increments in mean PD (CAL) of 1 mm were significantly associated with increases in fibrinogen serum levels of 0.13 (0.06) g/L and of WBC of 0.5 (0.24) × 10^9^ cells/L. In obese patients, such associations were less pronounced or not significant.
Meisel et al., 2014 [45]	Prospective cohort study	2.746 subjects, 1.337 men and 1.409 women, from SHIP	Obesity	PD, CAL	CRP, IL-6	Tooth loss appeared to be associated with BMI, WHR and CRP and IL-6 levels. This association was more distinctive in men than in women. Men with increased levels of CRP or IL-6 had higher risk of tooth loss compared to men with CRP ≤2 mg/L during the 5 year F-U period. The impact of elevated BMI on tooth loss was higher in men than in women, and the association of CRP in the evaluation attenuated the impact of elevated BMI on the risk of tooth loss in men but not in women.
Nibali et al., 2007 [46]	Case-control study	485 subjects, 302 with periodontitis, 183 controls	CVD	PD, CAL	WBC	Periodontitis patients exhibited a higher count of WBC, an inflammatory risk factor for CVD. Severe periodontitis patients displayed an increase in leukocytes, especially neutrophils and lymphocytes. The higher the number of deep pockets in these patients, the higher the leukocyte number.
Del Pinto et al., 2018 [47]	Review	NA	CVD, cerebrovascular disease, CHD, CKD	NA	TNF, IL-1, IL-6	Periodontitis is a source of chronic LGI. Accumulating evidence supports the existence of systemic effects of periodontal inflammation on cardiovascular risk factors and diseases. History of periodontitis has been associated with incident cerebrovascular disease, CHD, CKD and mortality. Periodontitis is associated with a worse systolic blood pressure profile during antihypertensive therapy. The accumulation of AGEs during periodontitis is one of the triggers to the cascade of pro-inflammatory signaling that also subsequently activates the release of cytokines (TNF, IL-1, IL-6) into the bloodstream. Circulating inflammatory mediators, in turn, elicit endothelial dysfunction, with consequent impaired vasodilation and alterations in the vascular structure.
Holmstrup et al., 2017 [40]	Review	NA	CVD, Parkinson’s disease	NA	IL-6, TNF-α	Periodontitis is more frequent in patients with AMI. Periodontitis and CVD have various explanatory models for their association and among them there is LGI, which could be caused by the spillover of cytokines from periodontal tissues to the bloodstream. Patients that received periodontal treatment had a significantly lower incidence of AMI and stroke. Periodontitis is more common in patients with Parkinson’s disease and systemic LGI induced by periodontitis contributes to neural dysfunction at early stages of Parkinson’s disease.
Gurav, 2014 [48]	Review	NA	Vascular endothelial dysfunction, CVD	NA	IL-1, IL-6, TNF-α, MMP, PGE2, CRP	Periodontal inflammation causes the expression of various proinflammatory markers (IL-1, IL-6, TNF-α, MMP, PGE2, CRP), that are spilled into the bloodstream eliciting a state of low-grade systemic inflammation that may culminate in endothelial dysfunction. TNF-α and IL-6 reduce the endothelial NO synthase production, limiting NO production, and, thus, causing endothelial dysfunction. Periodontal treatment reduces systemic inflammation and improves endothelial function.
Gurav, 2014 [49]	Review	NA	Alzheimer’s disease	NA	IL, TNF-α, PGE2, MMP	The pro-inflammatory markers produced during periodontitis and streamed through the ulcerated periodontal pocket into systemic circulation cause a systemic LGI. These pro-inflammatory molecules can compromise the blood brain barrier and gain access to the cerebral regions. This may result in activation of microglial cells and the adverse repercussions leading to neuronal damage.
Sima et al., 2013 [50]	Review	NA	Diabetes mellitus	NA	CRP, TNF-α, IL-6, fibrinogen	Uncontrolled diabetes mellitus correlates with higher prevalence, severity and progression rate of periodontitis. Severe periodontitis increases the risk of cardiovascular and renal complications in patients with diabetes mellitus. One plausible explanation for the link between periodontitis and glycemic control is LGI, measured as elevation in systemic pro-inflammatory markers. Periodontitis raises the levels of pro-inflammatory and pro-thrombotic mediators in serum, whereas periodontal treatment improves metabolic control by a significant reduction in HbA1c levels and reduction in circulating inflammatory mediators (CRP, TNF, IL-6 and fibrinogen).
Shrihari, 2012 [51]	Review	NA	CHD	NA	CRP	Periodontitis causes low increases in CRP levels (2–3 times higher, while the increases seen in acute infection are 100–1000 times higher), that may be the link to increased CVD risk. CRP increased levels associated with periodontitis are also linked to increased carotid wall thickness. Increasing severity of periodontitis creates a higher risk of AMI and hypertension development. The number of teeth lost due to periodontitis has a relationship with CHD. All the studies included in this review demonstrated that nonsurgical periodontal therapy led to a decrease in serum CRP, in conjunction with improved endothelial function, but one study did not show a reduction of CRP levels after periodontal treatment. Patients with better clinical responses to periodontal treatment resulted in a greater decrease in CRP levels and had a greater reduction of their CVD risk.
Santos Tunes et al., 2010 [52]	Review	NA	Type 2 diabetes	NA	IL-1, IL-6, TNF-α, PGE2, CRP, fibrinogen	Periodontitis determines higher levels of IL-1, IL-6, TNF-α, PGE2, CRP and fibrinogen through their direct flow from the periodontium to the systemic circulation and through their increased production by immune cells. These inflammatory factors determine a status of chronic LGI which may contribute to insulin resistance. Some studies included in this review showed that periodontal non-surgical treatment reduced circulating levels of TNF-α and HbA1c. Conversely, another study showed no significant reduction of TNF-α, while CRP and soluble E-selectin levels were significantly reduced. A meta-analysis of 10 intervention trials and a recent RCT included in this review showed that HbA1c levels decreased following periodontal treatment, but not in a statistically significant way, while other studies showed significant improvements in glycemic control with periodontal therapy.
Moutsopoulos et al., 2006 [53]	Review	NA	Atherosclerosis, AMI, diabetes	NA	IL-6, CRP	In periodontal patients, leukocyte counts, IL-6 and CRP have been shown to be slightly elevated compared to healthy subjects in relation to the extent of disease. In the studies included in this review, plasma CRP levels ranged from 2 to 10 mg/L, consistent with the presence of low-grade chronic inflammation. Periodontal therapy leads to a decrease in serum CRP and cytokines, especially in patients with the highest starting levels of CRP.
Endo et al., 2010 [54]	Animal study	24 rats, six lean without periodontitis (CG); six lean with periodontitis (PG); six obese without periodontitis (OG); six obese with experimental periodontitis (MG).	Obesity	Histological evaluation of the linear distance between cementoenamel junction and alveolar bone crest	TNF-α, CRP	At 4 weeks, the number of polymorphonuclear leukocytes was 3.1, 2.2 and 4.6 times higher in the PG, OG, and MG, respectively, than in the CG. In the liver, the gene expression for CRP, IL-6 and TNF-α in the OG and MG was significantly higher than in the CG and PG. The gene expression for CRP and TNF-α in the MG was significantly higher than that in the OG by 1.4 and 1.6 times, respectively. In WAT, the gene expression for CRP, IL-6 and TNF-α in the OG and MG was significantly higher than in the CG and PG. Furthermore, gene expression for CRP and IL-6 in the MG was significantly higher than in the OG by 1.7 and 1.9 times, respectively. The mean levels of serum TNF-α in the PG and OG were 5% and 10% higher, respectively, than that of the CG, and these differences were significant. Serum TNF-α in the MG was significantly higher than in the PG and OG. The mean serum levels of CRP in the OG and MG were 1.9 and 2.2 times higher, respectively, than that of the CG. The mean serum level of CRP in the MG was significantly higher than each of the three groups.

SLE-I: systemic lupus erythematosus-inactive; SLE-A: systemic lupus erythematosus-active; PD: pocket depth; CAL: clinical attachment level; BOP: bleeding on probing; MIP: monocyte/macrophage inflammatory protein; IL: Interleukin; IP: interferon gamma-induced protein; INF: interferon; GM-CSF: granulocyte-macrophage-colony-stimulating factor; TNF: tumor necrosis factor; MCP: monocyte chemoattractant protein; sICAM: soluble intercellular adhesion molecule; LGI: low-grade inflammation; SHIP: Study of Health In Pomerania; WBC: white blood cells; F-U: follow-up; CVD: cardiovascular disease; BMI: body mass index; WHR: waist-to-hip ratio; CRP: C-reactive protein; NA: not applicable; CHD: coronary heart disease; CKD: chronic kidney disease; AGE: advanced glycation end products; AMI: acute myocardial infarction; MMP: matrix metalloproteinase; PGE2: prostaglandin E2; NO: nitric oxide; HbA1c: hemoglobin A1c; RCT: randomized controlled trial; WAT: white adipose tissue.
